# Intestinal Microbial Diversity of Free-Range and Captive Yak in Qinghai Province

**DOI:** 10.3390/microorganisms10040754

**Published:** 2022-03-31

**Authors:** Ying Wen, Shaofei Li, Zishuo Wang, Hao Feng, Xiaoting Yao, Mingjie Liu, Jianjun Chang, Xiaoyu Ding, Huiying Zhao, Wentao Ma

**Affiliations:** 1 College of Agriculture and Animal Husbandry, Qinghai University, Xining 810016, China; changjianjun2020@163.com (J.C.); d13997094994@163.com (X.D.); 2 College of Veterinary Medicine, Northwest A&F University, Xianyang 712100, China; shaofeili@nwafu.edu.cn (S.L.); wangzishuo@nwafu.edu.cn (Z.W.); 99fen98hao1900@nwafu.edu.cn (H.F.); yaoxiaoting@nwafu.edu.cn (X.Y.); mreight@nwafu.edu.cn (M.L.)

**Keywords:** free-range yaks, captive yaks, feeding style, gut microbiota, sequencing

## Abstract

Background: The gut microbiome is a large and complex organic assemblage with subtle and close relationships with the host. This symbiotic mechanism is important for the health and adaptability of the host to the environment. Compared with other ruminants, there are few studies on yak intestinal microbes. The study of the gut microbiota of the yak will help us better understand the correlation between the microbiota and the environmental adaptability of the host. In this study, we adapted 16S rDNA sequencing technology to investigate the diversity and composition of the intestinal microbial community in free-range yaks and captive yaks living on the Qinghai–Tibet Plateau (QTP). Results: Sequencing results showed that the intestinal microbial community diversity was significantly different between free-range yaks and captive yaks. *Firmicutes* and *Bacteroidetes* were the dominant bacteria in both free-range and captive yaks. However, there were differences between the microbes of the two analyzed feeding styles in different classification levels. Compared with the captive type, free-range yaks had a higher abundance of *Ruminococcaceae*, *Eubacteriaceae*, *Desulfovibrionaceae*, *Elusimicrobium*, and *Oscillibacter*, while the abundance of *Succinivibrionaceae*, *Clostridiales*, *Lachnospiraceae*, *Prevotellaceae*, *Roseburia*, and *Barnesiella* was relatively low. The feeding method may be the key factor for the formation of intestinal flora differences in yaks, while altitude did not significantly affect Qinghai yak. Conclusions: In this study, we used 16S rDNA sequencing technology to investigate the composition of intestinal flora in free-range and captive yaks living on the QTP. The exploration of dietary factors can provide a theoretical basis for scientifically and rationally breeding yaks and provides a new direction for the development of prebiotics and microecological agents.

## 1. Introduction

The intestinal microbiota is an aggregate of millions of microbes that exist in the gastrointestinal tract, and it interacts extensively with the host. From the beginning of life until death, the gut microbiota exhibits varying characteristics among individuals and throughout their development, yet it maintains its relative stability and diversity [1]. The collective genes of the gut microbiota are 150 times larger than the human genome [2]. In recent years, intestinal flora has been regarded as a signal hub that impacts on the host’s metabolism, immunity, and infection response by integrating environmental inputs, genetic factors, and immune system signals [3]. Various factors can disrupt the balance and cause a state termed “dysbiosis” [4]. Studies have shown that the “dysbiosis” of microbial composition often has harmful effects on human health including the induction of type 2 diabetes, obesity, inflammatory bowel disease, and neurological diseases [5,6,7,8]. However, the intestinal microbial community of adults is in a relatively balanced condition with a degree of resistance to certain disturbances [9] such as antibiotics [10]. Therefore, it is necessary to study the factors that can change the composition of animals’ gut microbes. Excitingly, with the in-depth study of the microbial community, intestinal flora has gradually demonstrated its tremendous therapeutic potential for related diseases [11,12,13].

The abundance and diversity of the gut microbial community are affected by factors such as species, genotype, age, diet, sex, and a plethora of environmental parameters [14,15,16,17,18]. Environmental factors have more impact on shaping the host gut microbiome than genetic factors [19]. One of the most concerned factors is the influence of diet on the plasticity of the host gut microbiota. Diet participates in microbial metabolism, and the products of these physiological processes are inextricably linked with the health or disease development of the host [20]. In addition, the host’s exercise status can regulate the intestinal microbial profile and its metabolic level, enhance the host’s ability to take up nutrients, and improve insulin sensitivity [21]. By exploring the influence of these factors in the gut microbiota, it can help us better understand the relationship between microbes and health.

Yak is a characteristic animal living in Qinghai–Tibet Plateau (QTP) and has been domesticated by nomads for 7300 years [22]. Statistics show that there are more than 22 million domestic yaks in the QTP and surrounding high-altitude areas, providing local people with living resources such as meat, milk, and fur [23]. Yaks have numerous unique physiological traits that help them adapt to the environment at high altitudes [23]. Under traditional management, yaks graze on natural pastures year-round without the need for additional feed [24]. Studying the adaptation mechanism of the yak to the high-altitude environment may advance our understanding, treatment, and prevention of hypoxia-related diseases in humans and animals. In addition, yak is a type of livestock that can survive in environments of extreme cold, hypoxia, and limited grass resources, and they are an important economic pillar of animal husbandry of the QTP [25,26]. Exploring the relationship between the microbiota and the environmental adaptability of the yak from the perspective of the intestinal microbiome can provide some inspiration for scientific planning of yak breeding.

Previous studies have found that the gastrointestinal microbiota of ruminants maintains a good symbiotic relationship with the host [27,28]. Previous studies have confirmed that differences in feeding styles and altitude can significantly affect the structure of the rumen microbiota of yak [29,30], but the impact of these two factors on the gut microbiome needs to be further studied. In order to analyze the effects of different feeding styles on intestinal microbiota of yaks, we amplified the V3 and V4 regions of 16S rDNA in their stool samples and sequenced the amplified products on an Illumina MiSeq platform. We investigated the composition and structure of the gut microbiota of yaks. The results of this study will help to further understand the relationship between gut microbes and yak’s ability to adapt to the environment and provide new ideas for the development of prebiotics and microecological agents.

## 2. Materials and Methods

### 2.1. Sample Collection

A total of 38 yaks were divided based on feeding styles: free-range and captive breeding groups. Fecal samples were collected from two farms with different altitudes (~1000 m) in Qinghai Province and used for 16S rDNA sequencing (10 free-range yaks in Xinghai County (Fx), 8 free-range yaks in Haiyan County (Fh), 6 captive yaks in Xinghai County (Cx), and 14 captive yaks in Haiyan County (Ch)). Feces were collected from 7:00 to 9:00 in the morning. After the yaks were observed to excrete feces, sterile cotton swabs were immediately used to remove the external feces, and new sterile cotton swabs were used to collect part of the internal feces into sterile EP tubes, transported to the laboratory after being placed in liquid nitrogen, and stored at −80 °C. DNA was extracted after all samples were collected. The selected yaks were in a healthy state and did not suffer from other diseases before samples were collected. All yaks were of the same breed and were in the natural breeding conditions of local farmers and herders, and no antibiotic use was recorded. No intervention measures were taken except collecting fecal samples. Detailed information of the fecal sample is listed in Table S1.

### 2.2. DNA Extraction

The total DNA was extracted using MagPure Stool DNA KF Kit B (Magen, Guangzhou, China), and total DNA was quantified with a Qubit Fluorometer by using Qubit dsDNA BR Assay Kit (Invitrogen, Waltham, MA, USA) in accordance with the manufacturer’s instruction. Blank controls were set-up to confirm the authenticity of the sample DNA. In addition, the DNA’s quality was checked on 1% agarose gel and stored at −20 °C.

### 2.3. PCR Amplification and Library Construction

Broadly conserved primers, 341F (5′-ACTCCTACGGGAGGCAGCAG-3′) and 806R (5′-GGACTACHVGGGTWTCTAAT-3′) were used to amplify the 16S rRNA gene-specific variable regions V3 and V4 of bacteria [31]. An Illumina adapter was used to tag forward and reverse primers for further sequencing. All polymerase chain reactions (PCRs) were enriched in a 50 μL reaction a containing 30 ng template, fusion PCR primer, and 2×Phanta^®^ Max Master Mix (Vazyme, Nanjing, China, P515-01). The thermal cycling conditions were set as follows: 94 °C (3 min), 30 cycles of 94 (30 s), 56 (45 s), 72 (45 s), and 72 °C (10 min). The PCR reaction system was configured with 30 ng DNA and fusion primer. Amplification was carried out after setting reaction parameters. Meanwhile, no-template controls were set to ensure the specificity of PCR products. The PCR products were purified using AmpureXP beads and elution buffer, and after labeling, the library construction was finished. The library was qualified using an Agilent 2100 Bioanalyzer (Agilent, Santa Clara, CA, USA). The validated libraries were used for sequencing on an Illumina MiSeq platform (Illumina, San Diego, CA, USA) with the sequencing strategy MiSeq-PE300 (MiSeq-PE301+8+8+301). MiSeq Reagent Kits v3 (600 cycle) were used as cartridges for sequencing library pools.

### 2.4. Statistical Analysis

Cutadapt (v2.6) was used to filter the raw reads to the acquired high-quality clean reads [32]. After intercepting primer and joint contamination, the method of removing low quality by window was adopted. The window length was set to 30 bp, and if the average window quality value was less than 20, the read-end sequence was truncated from the window, and reads that had a final read length that was less than 75% of the original read length were removed. In addition, reads containing N and reads with low complexity (10 consecutive ATCG) were removed to obtain the final clean data. All filtered reads were imported into QIIME2 (v2020.2) [33], based on the method of DADA2 (Divisive Amplicon Denoising Algorithm), and the imported paired-end sequences were denoised using the QIIME DADA2 denoise-paired command to obtain amplicon sequence variants (ASVs) with 100% sequence similarity (the truncation length was set to 0, and the minimum overlap was set to 20 bp) [34]. Finally, the ASV representative sequences were compared with the database (Greengene: v201305) for species annotation by the Ribosomal Database Project (RDP) classifier (v1.9.1) software, and the confidence threshold was set to 0.6 [35]. RStudio (v4.0.2) was used to summarize and analyze the ASVs information. The “VennDiagram” package in R was performed to create the Venn diagram [36]. The “vegan” package in R was used to calculate the microbial alpha diversity index, Bray-Curtis distance, and unweighted UniFrac distance, which were used to evaluate the similarity of the different samples [37]. In addition, the phylogenetic tree was acquired by sequence comparison with FASTTREE (v2.0). The “Phyloseq” and “ggplot2” packages were used for principal coordinate analysis (PCoA) analysis and hierarchical cluster analysis [38,39]. The ALDEx2 package was applied for ASV differential abundance analysis [40,41,42]. The Wilcoxon test, PERMANOVA, and ANOSIM were used to evaluate significant differences between different groups.

## 3. Results

### 3.1. Sequencing Data

**Figure 1:**
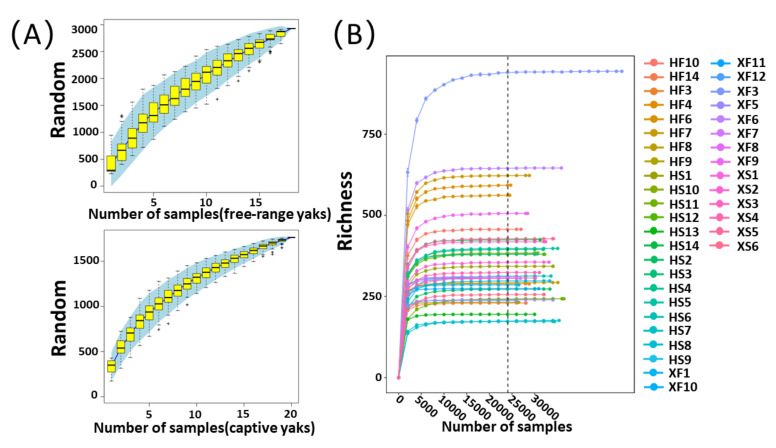
Species accumulation and dilution curves for all samples: (**A**) species accumulation curves of free-range and captive yak samples; (**B**) rarefaction curves of all samples. The legend is the number of all samples.

Thirty-eight fecal samples were collected in free-range yaks (10 yaks in Xinghai County (Fx) and eight yaks in Haiyan County (Fh)) and captive yaks (six yaks in Xinghai County (Cx) and 14 yaks in Haiyan County (Ch)). As mentioned above, clean reads with a length of 300 ± 3 bp were generated after sequencing in paired-end mode. The total amount of data for each sample was in the range between 60,601 × 2 and 71,920 × 2. The sequencing data for all of the samples are listed in Table S2. The species accumulation curves of the yak samples for the two feeding styles were relatively flat with the increase in the number of samples, indicating that the existing sample size could basically meet the needs of this study (Figure 1A). Rarefaction curves also indicated that the depth of sequencing was reasonable (Figure 1B). In order to avoid the impact of sequencing errors or random factors on the analysis results, ASVs with low abundance (i.e., total less than 10) were filtered, and the remaining ASVs were used for subsequent analysis.

### 3.2. Intestinal Flora Is Associated with Yak Feeding Styles

**Figure 2:**
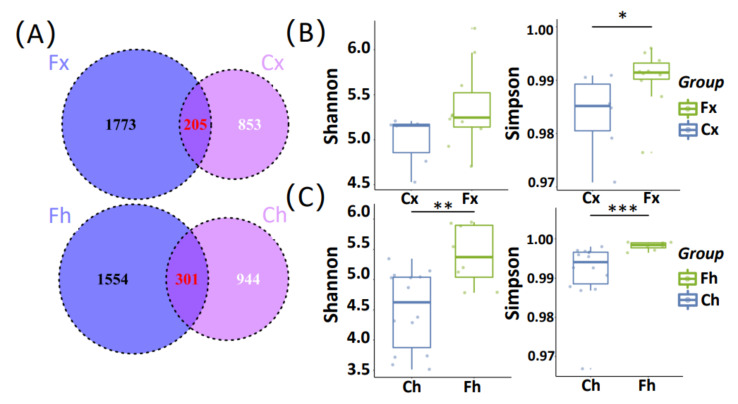
Analysis of the composition and diversity of intestinal microbes. Fx, free-range yaks in Xinghai County; Cx, captive yaks in Xinghai County; Fh, free-range yaks in Haiyan County; Ch, captive yaks in Haiyan County. (**A**) Venn diagram showing the OTU overlap of Fx and Cx and Fh and Ch, respectively; (**B**) Shannon index and Simpson index of Fx and Cx; (**C**) Shannon index and Simpson index of Fh and Ch. Statistical test results are represented by *p*-value (* *p* < 0.05, ** *p* < 0.01, *** *p* < 0.001).

After cleaning the raw data, 1978, 1058, 1855, and 1245 ASVs were obtained from samples for groups Fx, Cx, Fh, and Ch, respectively (Table S2). A total of 4268 ASVs were detected in all samples, of which 205 (6.75% of all ASVs in Xinghai County) and 301 (9.71% of all ASVs in Haiyan County) were core ASVs (Figure 2A). Furthermore, 1773 and 853 unique ASVs were detected in the Fx and Cx groups, and 1554 and 944 ASVs were uniquely found in the Fh and Ch groups.

In order to determine whether the different feeding styles had a significant impact on the intestinal microbial diversity of the yaks, we tested the α-diversity and β-diversity of the yak gut microbial fraction. The Shannon index of the Fh group, Cx group, Fh group, and Ch group were 5.35, 5.00, 5.40, and 4.50, respectively. Moreover, the Simpson index (Simpson’s index of Diversity 1-D) were 0.990, 0.984, 0.991, and 0.958, respectively. Tables S3 and S4 list the α-diversity index of the four groups of samples. In general, there was a significant difference in the α-diversity index between free-range yaks and captive yaks. Figure 2B,C show the Shannon index and Simpson index of the two feeding styles of yaks in Xinghai County and Haiyan County, respectively. The Shannon index for the Fh group was significantly higher than for the Ch group (Wilcox, *p* < 0.01), and the Simpson index for the Fx and Fh groups were significantly higher than for the Cx and Ch groups (Wilcox, *p* < 0.05 and *p* < 0.001). In other words, the Fx and Fh groups showed higher microbial diversity than the Cx and Ch groups. It also indicated that the difference of intestinal microflora composition of yaks was related to feeding styles.

### 3.3. Comparison of Gut Microbial Diversity between Free-Range Yaks and Captive Yaks

**Figure 3:**
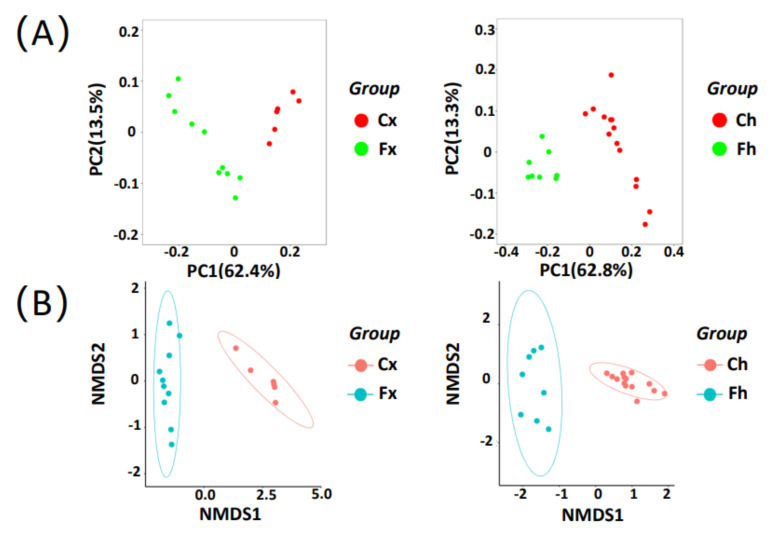
Analysis of the intestinal flora composition of free-range yaks and captive yaks: (**A**) principal component analysis (PCoA) based on unweighted UniFrac distance shows the distribution between samples of different groups; (**B**) nonmetric multidimensional scaling (NMDS) based on Bray-Curtis distance analyzed the microbiome similarity of samples. PC1, PC2, NMDS1, and NMDS2 are the first two principal components in dimension reduction of the sample data, respectively.

The ASVs’ abundance information of the sample was used to calculate the Bray-Curtis distance matrix. Based on evolutionary distance, UniFrac was performed to evaluate the similarity of samples between groups. The results of unweighted UniFrac principal component analysis (PCoA) showed that there were significant differences among yaks with different feeding styles in Figure 3A (PERMANOVA, *p* = 0.001). All the samples were divided into two clusters, and the scattered points indicated the relationship between free-range and captivity. The principal components, PC1 and PC2, of the Fx group and the Cx group accounted for 62.4% and 13.5% of the explained variance; moreover, the principal components of the Fh group and the Ch group, respectively, accounted for 62.8% and 13.3%. The results showed that there were significant differences in the composition of the intestinal microflora between free-range yaks and captive yaks (PERMANOVA, *p* = 0.001).

Nonmetric multidimensional scaling (NMDS) was performed to assess the similarity of Bray-Curtis to illustrate the differences in microbial populations among the groups, according to the ASVs’ abundance in the samples. The Fx group and Cx groups showed obvious separate clustering in Figure 3B (ANOSIM, *p* = 0.001), and the results of the Fh group and Ch group were consistent with the former (ANOSIM, *p* = 0.001).

**Figure 4:**
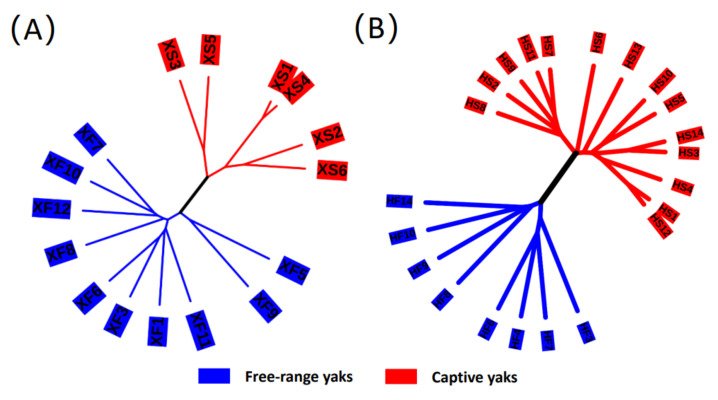
Hierarchical cluster analysis of microbial communities for all samples based on Bray-Curtis distance: (**A**) samples from Xinghai County; (**B**) sample from Haiyan County. The number is the ID of the sample.

In addition, we adopted hierarchical clustering analysis to explore the similarity among all samples. UPGMA and Bray-Curtis distance similarity were used for cluster analysis (Figure 4). All samples were divided into two clusters: one included all the free-range group samples (i.e., FX and Fh) and the other consisted of all the captive group samples (i.e., Cx and Ch). The differences between the samples from the free-range group and the captive group were significant, which was consistent with the above results. Therefore, we speculated that feeding styles could greatly influence the composition of intestinal microflora of yaks.

### 3.4. Community Composition of Intestinal Microbes in Different Feeding Methods

**Figure 5:**
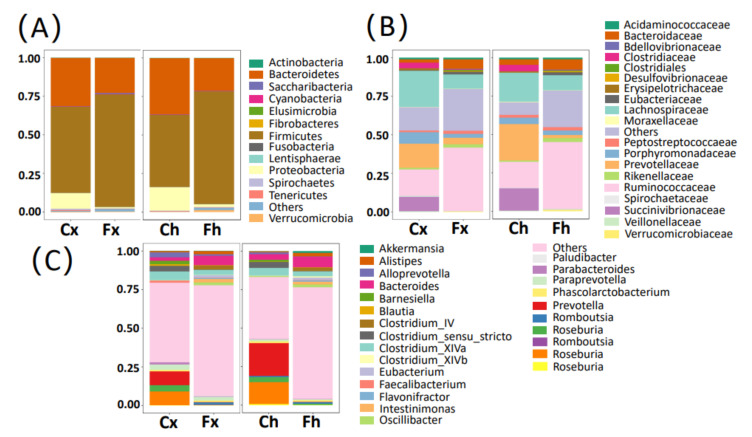
Intestinal microbiome composition of samples from different groups. The top 15 taxa with high abundance were selected to calculate the relative abundance to form a stack map: (**A**) composition of microbiota at the phylum level; (**B**) composition of microbiota at the family level; (**C**) composition of microbiota at the genus level.

Based on the ASVs’ annotation information for all samples, the intestinal microbiome composition between free-range yaks and captive yaks from the same farm at different classification levels was statistically analyzed so as to eliminate the interference of regional factors on the results. Figure 5A shows the bacterial composition at the phylum level of intestinal microorganisms between free-range and captive yaks in Xinghai County and Haiyan County, respectively. The dominant phylum in all samples were *Firmicutes* and *Bacteroidetes*. In addition, the abundance of *Proteobacteria* was also high. However, compared with Cx and Ch, the abundance of *Firmicutes* and *Proteobacteria* in Fx and Fh was higher (Wilcox, *p* < 0.05), while *Lentisphaerae* and *Fibrobacteres* were not found in Cx and Ch.

The bacterial composition of the different flora at the family level is presented in Figure 5B. As shown in Figure 5B, *Ruminococcaceae* was the most abundant dominant family in Fx and Fh, while *Succinivibrionaceae* was almost nonexistent. *Lachnospiraceae* and *Prevotellaceae* were the dominant families with the highest abundance among the Cx and Ch, while *Eubacteriaceae* and *Desulfovibrionaceae* hardly existed. In comparison, the abundance of *Ruminococcaceae, Eubacteriaceae*, and *Desulfovibrionaceae* in Fx and Fh was higher (Wilcox, *p* < 0.01), while the abundance of *Succinivibrionaceae*, *Clostridiales*, *Lachnospiraceae*, and *Prevotellaceae* were significantly lower than Cx and Ch (Wilcox, *p* < 0.01).

The bacterial compositions of Fx and Fh were significantly different from Cx and Ch at the genus level (Figure 5C). Table 1 and Table S5 show the relative abundance of the bacterial composition of yaks in the free-range group and captive group. *Bacteroides* was the dominant genera in Fx and Fh, while *Succinivibrio* and *Prevotella* were the main genera in Cx and Ch. *Elusimicrobium* and *Oscillibacter* in Fx and Fh were higher than those of Cx and Ch (Wilcox, *p* < 0.01), while *Roseburia* and *Barnesiella* were much lower (Wilcox, *p* < 0.01). In addition, *Clostridium_IV*, *Intestinimonas*, and *Eubacterium* were almost absent in the Cx and Ch groups.

## 4. Discussion

In this study, 16S rDNA sequencing was used to assess the intestinal microbial composition of yaks with two feeding styles. We calculated and compared the diversity and abundance of free-range and captive yaks in two farms with 1000 m difference in altitude of Qinghai Province. Differential abundance analysis showed that there were significant differences between the free-range groups (i.e., Fx and Fh) and captive groups (i.e., Cx and Ch) in Figure S1A,B. Previous studies have shown that different feeding styles or sudden dietary changes can significantly affect the intestinal microbiota structure of ruminants [43,44,45,46], which is consistent with our results. In order to determine whether different rearing methods had significant effects on the intestinal microbiota of yaks, we evaluated the α-diversity, β-diversity, and hierarchical clustering of the intestinal microbiota in captive and free-range yaks. These results suggest that there are indeed differences in the intestinal microbiota under different feeding styles. Furthermore, the intestinal flora of yaks with two feeding styles changed at different taxonomic levels. Considering that all the samples came from different altitudes, we evaluated the effects of altitude on the intestinal microbiota of yaks and found that there was no significant difference between the samples with the same feeding style (Figure S2). In addition, differential abundance analysis revealed no significantly different taxa of captive yaks from the two farms (Figure S1C). This also means that altitude has no significant effect on the intestinal microbes of Qinghai yaks. The complexity of the external environment may be the reason for the small number of distinct taxa in free-range yak samples (Figure S1D). Therefore, we speculated that the dietary composition of captive yaks is more regular and stable due to the artificial intervention, which may be related to the relatively conservative nature of intestinal flora of captive yaks. There are many factors in the living environment of free-range yaks such as climate, soil, and temperature. These environmental variables could affect the dietary structure of yaks. Changes in the composition of the gut microbiome may help the host adjust its metabolism and meet its needs, helping the host better adapt to the high-altitude environment. However, there are several limitations of the present study. For example, we did not deliberately control for the sex and age of the yaks when sampling. Therefore, the influence of intestinal microbiota by sex and age could not be ruled out in this study.

*Firmicutes* and *Bacteroidetes* are currently known to be the most abundant at the phylum level in the gastrointestinal microbiota of ruminants [43,47,48,49,50,51]. Among all the samples in this study, the abundance of *Firmicutes* and *Bacteroidetes* of yaks in Xinghai County accounted for 56.59% and 31.03% of the total microorganisms and 66.68% and 26.16% of the total microbes in Haiyan County yak, which were the most dominant phyla. These results are consistent with several previous studies [43,47,48,49,50,51]. *Firmicutes* in the gastrointestinal tract have previously been reported to effectively decompose cellulose and lignin [46,52]. *Bacteroidetes* have the function of decomposing non-fiber complex polysaccharides and maintaining intestinal balance [53]. Both are closely related to the metabolism of fiber and non-fiber food components of the host. In addition, we found that *Proteobacteria* also occupied a high relative abundance in free-range yaks. *Proteobacteria* are reported to be the core flora in ruminant digestion of soluble carbohydrates and are also involved in host biofilm formation and gastric content fermentation [54,55]. The high abundance of *Firmicutes* and *Proteobacteria* means that the gut microbes of the free-range yak are more capable of utilizing gut contents.

The abundance ratio of *Firmicutes* and *Bacteroidetes* changed between the two feeding styles. Therefore, we further studied the relative abundance composition of bacteria at the genus level. The results showed that the abundance of *Prevotella* in the free-range groups were significantly lower than that of the captive groups in *Bacteroidetes*, while the abundance of *Clostridium_IV*, *Intestinimonas*, and *Oscillibacter* in the free-range groups were relatively higher. The latter three belong to *Ruminococcaceae*. Some bacteria of *Ruminococcaceae* are rich in cellulase and xylanase and have strong hydrolysis capacity of polysaccharides [56]. *Prevotella* is representative in the gastrointestinal tract of vegetarian animals [57]. Studies have shown that *Prevotella* can decompose a variety of polysaccharides, maintain the energy balance of the bacterial community [58], and play a crucial role in the rumen digestion of plant fiber and the amino acid metabolism of ruminants [53,59]. *Clostridium_IV* is an important group of intestinal bacteria that produce butyric acid and other short-chain fatty acids (SCFAs) [60,61]. SCFAs are important energy materials and can be absorbed by the intestinal wall through ion exchange and passive diffusion to meet the caloric needs of the host [62]. It has been found that the intestinal butyrate level of the host is positively correlated with the content of *Clostridium_IV* and *Oscillibacter* [44,61]. Acetate, propionate, and butyrate are important components of SCFAs. Acetate is involved in lipid synthesis, and butyrate can provide energy for intestinal wall cells, prevent autophagy, and stimulate endocrine cells to secrete leptin to regulate intestinal energy balance [63,64].

A study of Mongolian sheep found that compared with grazing sheep, *Prevotella* is more abundant in the intestines of captive sheep, while *Succinivibrio* in the grazing group was not detected [44], which is similar to our results. Moreover, the researchers found that feeding goats with high-grain forage increased the abundance of *Prevotella* in the cecum [65]. *Succinivibrio* can decompose starch and provide energy by converting succinic acid into propionic acid for the host to absorb [52]. We hypothesized that dietary differences contribute to changes in the intestinal flora of Qinghai yaks. The adjustment of intestinal microbial structure in captive yaks could help them digest and absorb high carbohydrate feed better (mostly grain), while the rich cellulose and hemicellulose xylan in herbage may require a strong fiber digestion ability in free-range yaks. A high-fiber diet can produce a large number of SCFAs under the decomposition of intestinal flora and cause a decrease in local microenvironmental pH [66,67]. A decrease in pH has a selective effect on the microorganisms in the colon, resulting in an increase in the number of butyrate producing Gram-positive bacteria and a decrease in some Bacteroidetes [68]. Local pH may impact on the process of dietary factors causing differences in the intestinal microbial community. At the same time, the high concentration of SCFAs and low pH produced by microorganisms could inhibit the colonization and growth of pathogenic bacteria and potentially pathogenic microorganisms to a certain extent [18,62,66]. These parameters may protect yaks in pastoral areas against pathogens.

One study of mice found that the gut microbiome was altered in response to cold stimuli, most notably a decline in *Akkermansia* abundance, which led to a series of plasticity changes in the gut in response to increased energy demands [69]. However, we found an interesting phenomenon in our study: *Akkermansia* was almost not detected in yaks living in the barns (only two samples in the Ch group had extremely low abundance), while it was relatively high in yaks living in the cold free-range area at high altitude (Table S5, *p* < 0.05). A reduction in *Akkermansia* increases intestinal sensitivity to insulin, causes fat browning, and increases intestinal tract and intestinal villi length [69]. Maintaining the body temperature of resting animals in cold environments by enhancing nutrient absorption and metabolism and generating heat from brown fat decomposition seems to explain why *Akkermansia* was almost absent in captive yaks. Furthermore, we assumed that the motion state of free-range yaks could regulate the intestinal microbiota spectrum and the corresponding metabolic level, and the heat generated by the movement could meet their own needs or resist the invasion of low temperature in other ways after a long time of adaptation. Some species of *Akkermansia*, such as *Akkermansia muciniphila*, is involved in maintaining the integrity of the intestinal barrier of the host, inducing the differentiation of intestinal regulatory T cells and T follicular helper cells to regulate intestinal homeostasis [70,71,72]. This may enable free-range yaks to cope with environmental disturbances better. In this study, we found that there were significant differences in intestinal microbes of Qinghai yaks with different feeding methods. However, there was no significant difference in the intestinal microbiota of free-range and captive yaks with altitude difference of nearly 1000 m, which attracted our attention. Is this relatively stable phenomenon related to yaks’ compensatory mechanisms such as cold resistance and hypoxia resistance? If they are related, can we use intestinal microorganisms as mediators to regulate this compensatory mechanism of the host? These results suggest that the mechanism in this area needs further research.

## 5. Conclusions

We investigated the composition of the intestinal bacteria in free-range yaks and captive yaks and described their difference. The structure and diversity of the intestinal microflora of yaks were changed by different feeding methods, and this effect may not be restricted by altitude. In this study, we also found that changes in the composition or abundance of certain bacteria may be related to host health and adaptation to the external environment. Studying the effect of these factors on the intestinal microbial community can help us further understand the interaction mechanism between microbes and the host, provide reference for better rearing of yaks, and also provide a new prospective for the development of prebiotics and microecological agents.

## Figures and Tables

**Table 1:** The relative abundance of the first 10 genera expressed as the percentage of the total microbial abundance of free-range yaks (*n* = 18) and captive yaks (*n* = 20).

Genera	Group		Genera	Group	
	Fx (Mean)	Fh (Mean)		Cx (Mean)	Ch (Mean)
*Bacteroides*	6.31%	6.92%	*Prevotella*	8.89%	21.21%
*Clostridium_XlVa*	3.52%	3.51%	*Succinivibrio*	9.22%	14.27%
*Clostridium_IV*	2.91%	2.42%	*Clostridium_XlVa*	5.80%	5.21%
*Alistipes*	2.07%	2.40%	*Clostridium_sensu_stricto*	3.62%	4.06%
*Eubacterium*	1.78%	1.84%	*Bacteroides*	2.26%	3.75%
*Intestinimonas*	2.30%	1.84%	*Roseburia*	3.90%	3.07%
*Oscillibacter*	1.64%	1.69%	*Paraprevotella*	3.48%	1.42%
*Romboutsia* *Akkermansia*	1.43% -	1.26% 1.16%	*Barnesiella* *Romboutsia*	2.36% -	1.21% 1.09%
*Phascolarctobacterium* *Paraprevotella*	1.03% 2.39%	0.91% -	*Phascolarctobacterium* *Alloprevotella*	- 3.10%	1.07% -
			*Faecalibacterium*	1.40%	-

## Data Availability

The sequence data reported in this study were deposited in the Genome Sequence Archive (Genomics, Proteomics, and Bioinformatics 2021) [73] at the National Genomics Data Center (Nucleic Acids Res 2022) [74], China National Center for Bioinformation/Beijing Institute of Genomics, Chinese Academy of Sciences (GSA: CRA006134).

## Supplementary Materials

The following supporting information can be downloaded at: https://www.mdpi.com/article/10.3390/microorganisms10040754/s1, Table S1: Sample information of yaks; Table S2: Sequence information of the sample; Table S3: Microbial sequence diversity and richness index of Yaks in Xinghai County; Table S4: Microbial sequence diversity and richness index of Yaks in Haiyan County. Table S5: The relative abundance of all taxa, Figure S1: Differential abundance analysis of different groups., Figure S2: α and β diversity of yaks in the same feeding style.

## References

[1] Adak A., Khan M.R. (2019). An insight into gut microbiota and its functionalities. Cell. Mol. Life Sci..

[2] Qin J., Li R., Raes J., Arumugam M., Burgdorf K.S., Manichanh C., Nielsen T., Pons N., Levenez F., Yamada T. (2010). A human gut microbial gene catalogue established by metagenomic sequencing. Nature.

[3] Thaiss C.A., Zmora N., Levy M., Elinav E. (2016). The microbiome and innate immunity. Nature.

[4] Belizário J.E., Faintuch J., Silvestre R., Torrado E. (2018). Microbiome and Gut Dysbiosis. Metabolic Interaction in Infection.

[5] Scheithauer T.P.M., Rampanelli E., Nieuwdorp M., Vallance B.A., Verchere C.B., Van Raalte D.H., Herrema H. (2020). Gut Microbiota as a Trigger for Metabolic Inflammation in Obesity and Type 2 Diabetes. Front. Immunol..

[6] Franzosa E.A., Sirota-Madi A., Avila J., Fornelos N., Haiser H.J., Reinker S., Vatanen T., Hall A.B., Mallick H., McIver L.J. (2019). Author Correction: Gut microbiome structure and metabolic activity in inflammatory bowel disease. Nat. Microbiol..

[7] Wang X., Sun G., Feng T., Zhang J., Huang X., Wang T., Xie Z., Chu X., Yang J., Wang H. (2019). Sodium oligomannate therapeutically remodels gut microbiota and suppresses gut bacterial amino acids-shaped neuroinflammation to inhibit Alzheimer’s disease progression. Cell. Res..

[8] Mangiola F., Ianiro G., Franceschi F., Fagiuoli S., Gasbarrini G., Gasbarrini A. (2016). Gut microbiota in autism and mood disorders. World J. Gastroenterol..

[9] Sommer F., Anderson J.M., Bharti R., Raes J., Rosenstiel P. (2017). The resilience of the intestinal microbiota influences health and disease. Nat. Rev. Microbiol..

[10] Palleja A., Mikkelsen K.H., Forslund S.K., Kashani A., Allin K.H., Nielsen T., Hansen T.H., Liang S., Feng Q., Zhang C. (2018). Recovery of gut microbiota of healthy adults following antibiotic exposure. Nat. Microbiol..

[11] Durack J., Lynch S.V. (2019). The gut microbiome: Relationships with disease and opportunities for therapy. J. Exp. Med..

[12] Caballero S., Kim S., Carter R.A., Leiner I.M., Sušac B., Miller L., Kim G.J., Ling L., Pamer E.G. (2017). Cooperating Commen-sals Restore Colonization Resistance to Vancomycin-Resistant Enterococcus faecium. Cell Host Microbe.

[13] Illiano P., Brambilla R., Parolini C. (2020). The mutual interplay of gut microbiota, diet and human disease. FEBS J..

[14] Davenport E., Cusanovich D., Michelini K., Barreiro L., Ober C., Gilad Y. (2015). Genome-Wide Association Studies of the Human Gut Microbiota. PLoS ONE.

[15] Andeweg S.P., Keşmir C., Dutilh B.E. (2021). Quantifying the Impact of Human Leukocyte Antigen on the Human Gut Microbiota. mSphere.

[16] Markle J.G.M., Frank D.N., Mortin-Toth S., Robertson C.E., Feazel L.M., Rolle-Kampczyk U., von Bergen M., McCoy K.D., Macpherson A.J., Danska J.S. (2013). Sex Differences in the Gut Microbiome Drive Hormone-Dependent Regulation of Autoimmunity. Science.

[17] Maynard C., Weinkove D., Harris J.R., Korolchuk V.I. (2018). The Gut Microbiota and Ageing. Biochemistry and Cell Biology of Ageing: Part I Biomedical Science.

[18] Bibbò S., Ianiro G., Giorgio V., Scaldaferri F., Masucci L., Gasbarrini A., Cammarota G. (2016). The role of diet on gut microbiota composition. Eur. Rev. Med. Pharmacol. Sci..

[19] Rothschild D., Weissbrod O., Barkan E., Kurilshikov A., Korem T., Zeevi D., Costea P.I., Godneva A., Kalka I.N., Bar N. (2018). Environment dominates over host genetics in shaping human gut microbiota. Nature.

[20] Gentile C.L., Weir T.L. (2018). The gut microbiota at the intersection of diet and human health. Science.

[21] Motiani K.K., Collado M.C., Eskelinen J.-J., Virtanen K.A., Löyttyniemi E., Salminen S., Nuutila P., Kalliokoski K.K., Hannukainen J.C. (2020). Exercise Training Modulates Gut Microbiota Profile and Improves Endotoxemia. Med. Sci. Sports Exerc..

[22] Liu Y., Luo J., Dou J., Yan B., Ren Q., Tang B., Wang K., Qiu Q. (2020). The sequence and de novo assembly of the wild yak genome. Sci. Data.

[23] Qiu Q., Zhang G., Ma T., Qian W., Wang J., Ye Z., Cao C., Hu Q., Kim J., Larkin D.M. (2012). The yak genome and adaptation to life at high altitude. Nat. Genet..

[24] Shi F., Wang H., Degen A.A., Zhou J., Guo N., Mudassar S., Long R. (2019). Rumen parameters of yaks (*Bos grunniens*) and indigenous cattle (*Bos taurus*) grazing on the Qinghai-Tibetan Plateau. J. Anim. Physiol. Anim. Nutr..

[25] Qiu Q., Wang L., Wang K., Yang Y., Ma T., Wang Z., Zhang X., Ni Z., Hou F., Long R. (2015). Yak whole-genome resequencing reveals domestication signatures and prehistoric population expansions. Nat. Commun..

[26] Zhang X., Wang K., Wang L., Yang Y., Ni Z., Xie X., Shao X., Han J., Wan D., Qiu Q. (2016). Genome-wide patterns of copy number variation in the Chinese yak genome. BMC Genom..

[27] Newbold C.J., Ramos-Morales E. (2020). Review: Ruminal microbiome and microbial metabolome: Effects of diet and ruminant host. Animal.

[28] Cholewińska P., Czyz K., Nowakowski P., Wyrostek A. (2020). The microbiome of the digestive system of ruminants—A review. Anim. Health Res. Rev..

[29] Huang C., Ge F., Yao X., Guo X., Bao P., Ma X., Wu X., Chu M., Yan P., Liang C. (2021). Microbiome and Metabolomics Reveal the Effects of Different Feeding Systems on the Growth and Ruminal Development of Yaks. Front. Microbiol..

[30] Wu D., Vinitchaikul P., Deng M., Zhang G., Sun L., Wang H., Gou X., Mao H., Yang S. (2021). Exploration of the effects of altitude change on bacteria and fungi in the rumen of yak (*Bos grunniens*). Arch. Microbiol..

[31] Beikler T., Bunte K., Chan Y., Weiher B., Selbach S., Peters U., Klocke A., Watt R.M., Flemmig T.F. (2021). Oral Microbiota Transplant in Dogs with Naturally Occurring Periodontitis. J. Dent. Res..

[32] Martin M. (2011). Cutadapt removes adapter sequences from high-throughput sequencing reads. EMBnet. J..

[33] Hall M., Beiko R.G. (2018). 16S rRNA Gene Analysis with QIIME2. Methods Mol. Biol..

[34] Callahan B.J., Mcmurdie P.J., Rosen M.J., Han A.W., Johnson A.J.A., Holmes S.P. (2016). DADA_2_: High-resolution sample inference from Illumina amplicon data. Nat. Methods.

[35] Cole J.R., Wang Q., Fish J.A., Chai B., McGarrell D.M., Sun Y., Brown C.T., Porras-Alfaro A., Kuske C.R., Tiedje J.M. (2014). Ribosomal Database Project: Data and tools for high throughput rRNA analysis. Nucleic Acids Res..

[36] Chen H., Boutros P.C. (2011). VennDiagram: A package for the generation of highly-customizable Venn and Euler diagrams in R. BMC Bioinform..

[37] Oksanen J., Blanchet F.G., Friendly M., Kindt R., Legendre P., McGlinn D., Minchin P.R., O’Hara R.B., Simpson G.L., Solymos P. (2020). Vegan: Community Ecology Package. https://CRAN.R-project.org/package=vegan.

[38] McMurdie P.J., Holmes S. (2013). phyloseq: An R Package for Reproducible Interactive Analysis and Graphics of Microbiome Census Data. PLoS ONE.

[39] Wickham H. (2016). ggplot2: Elegant Graphics for Data Analysis.

[40] Fernandes A.D., Macklaim J.M., Linn T.G., Reid G., Gloor G.B. (2013). ANOVA-Like Differential Expression (ALDEx) Analysis for Mixed Population RNA-Seq. PLoS ONE.

[41] Fernandes A.D., Reid J.N., Macklaim J.M., McMurrough T.A., Edgell D.R., Gloor G.B. (2014). Unifying the analysis of high-throughput sequencing datasets: Characterizing RNA-seq, 16S rRNA gene sequencing and selective growth experiments by compositional data analysis. Microbiome.

[42] Gloor G.B., Macklaim J.M., Fernandes A.D. (2016). Displaying Variation in Large Datasets: Plotting a Visual Summary of Effect Sizes. J. Comput. Graph. Stat..

[43] Chi X., Gao H., Wu G., Qin W., Song P., Wang L., Chen J., Cai Z., Zhang T. (2019). Comparison of gut microbiota diversity between wild and captive bharals (*Pseudois nayaur*). BMC Vet. Res..

[44] Wang B., Luo Y., Su R., Yao D., Hou Y., Liu C., Du R., Jin Y. (2020). Impact of feeding regimens on the composition of gut microbiota and me-tabolite profiles of plasma and feces from Mongolian sheep. J. Microbiol..

[45] Wang B., Luo Y., Wang Y., Wang D., Hou Y., Yao D., Tian J., Jin Y. (2021). Rumen bacteria and meat fatty acid composition of Sunit sheep reared under different feeding regimens in China. J. Sci. Food Agric..

[46] Zhang X.-L., Xu T.-W., Wang X.-G., Geng Y.-Y., Liu H.-J., Hu L.-Y., Zhao N., Kang S.-P., Zhang W.-M., Xu S.-X. (2020). The Effect of Transitioning between Feeding Methods on the Gut Microbiota Dynamics of Yaks on the Qinghai–Tibet Plateau. Animal.

[47] Zhong S., Ding Y., Wang Y., Zhou G., Guo H., Chen Y., Yang Y. (2019). Temperature and humidity index (THI)-induced rumen bacterial community changes in goats. Appl. Microbiol. Biotechnol..

[48] Mamun M.A.A., Sandeman M., Rayment P., Brook-Carter P., Scholes E., Kasinadhuni N., Piedrafita D., Greenhill A.R. (2020). The composition and stability of the faecal microbiota of Merino sheep. J. Appl. Microbiol..

[49] Guo J., Li P., Zhang K., Zhang L., Wang X., Li L., Zhang H. (2020). Distinct Stage Changes in Early-Life Colonization and Acquisition of the Gut Microbiota and Its Correlations with Volatile Fatty Acids in Goat Kids. Front. Microbiol..

[50] Faniyi T.O., Adegbeye M.J., Elghandour M., Pilego A.B., Salem A.Z., Olaniyi T.A., Adediran O., Adewumi M.K. (2019). Role of diverse fermentative factors towards microbial community shift in ruminants. J. Appl. Microbiol..

[51] O’Donnell M.M., Harris H.M.B., Ross R.P., O’Toole P.W. (2017). Core fecal microbiota of domesticated herbivorous ruminant, hindgut fermenters, and monogastric animals. MicrobiologyOpen.

[52] Zhang H., Shao M., Huang H., Wang S., Ma L., Wang H., Hu L., Wei K., Zhu R. (2018). The Dynamic Distribution of Small-Tail Han Sheep Microbiota across Different Intestinal Segments. Front. Microbiol..

[53] Gong G., Zhou S., Luo R., Gesang Z., Suolang S. (2020). Metagenomic insights into the diversity of carbohydrate-degrading enzymes in the yak fecal microbial community. BMC Microbiol..

[54] Qiu Q., Gao C., Gao Z., Rahman M.A.U., He Y., Cao B., Su H. (2019). Temporal Dynamics in Rumen Bacterial Community Composition of Finishing Steers during an Adaptation Period of Three Months. Microorganisms.

[55] Pitta D.W., Pinchak W.E., Indugu N., Vecchiarelli B., Sinha R., Fulford J.D. (2016). Metagenomic Analysis of the Rumen Microbiome of Steers with Wheat-Induced Frothy Bloat. Front. Microbiol..

[56] Mavrommatis A., Skliros D., Flemetakis E., Tsiplakou E. (2021). Changes in the Rumen Bacteriome Structure and Enzymatic Activities of Goats in Response to Dietary Supplementation with *Schizochytrium* spp.. Microorganisms.

[57] Holman D.B., Gzyl K.E. (2019). A meta-analysis of the bovine gastrointestinal tract microbiota. FEMS Microbiol. Ecol..

[58] Gálvez E.J., Iljazovic A., Amend L., Lesker T.R., Renault T., Thiemann S., Hao L., Roy U., Gronow A., Charpentier E. (2020). Distinct Polysaccharide Utilization Determines Interspecies Competition between Intestinal *Prevotella* spp.. Cell Host Microbe.

[59] Xue M.-Y., Sun H.-Z., Wu X.-H., Liu J.-X., Guan L.L. (2020). Multi-omics reveals that the rumen microbiome and its metabolome together with the host metabolome contribute to individualized dairy cow performance. Microbiome.

[60] Bobin-Dubigeon C., Bard J.-M., Luu T.-H., Le Vacon F., Nazih H. (2020). Basolateral Secretion from Caco-2 Cells Pretreated with Fecal Waters from Breast Cancer Patients Affects MCF7 Cell Viability. Nutrients.

[61] Zhu L.-B., Zhang Y.-C., Huang H.-H., Lin J. (2021). Prospects for clinical applications of butyrate-producing bacteria. World J. Clin. Pediatr..

[62] Martin-Gallausiaux C., Marinelli L., Blottière H.M., Larraufie P., Lapaque N. (2021). SCFA: Mechanisms and functional importance in the gut. Proc. Nutr. Soc..

[63] Nicholson J.K., Holmes E., Kinross J., Burcelin R., Gibson G., Jia W., Pettersson S. (2012). Host-Gut Microbiota Metabolic Interactions. Science.

[64] Rooks M.G., Garrett W.S. (2016). Gut microbiota, metabolites and host immunity. Nat. Rev. Immunol..

[65] Ye H., Liu J., Feng P., Zhu W., Mao S. (2016). Grain-rich diets altered the colonic fermentation and mucosa-associated bacterial communities and induced mucosal injuries in goats. Sci. Rep..

[66] Zimmer J., Lange B.J., Frick J.-S., Sauer H., Zimmermann K., Schwiertz A., Rusch K.A., Klosterhalfen S., Enck P. (2012). A vegan or vegetarian diet substantially alters the human colonic faecal microbiota. Eur. J. Clin. Nutr..

[67] Mueller N.T., Zhang M., Juraschek S.P., Miller E.R., Appel L.J. (2020). Effects of high-fiber diets enriched with carbohydrate, protein, or unsaturated fat on circulating short chain fatty acids: Results from the OmniHeart randomized trial. Am. J. Clin. Nutr..

[68] Duncan S.H., Louis P., Thomson J.M., Flint H.J. (2009). The role of pH in determining the species composition of the human colonic microbiota. Environ. Microbiol..

[69] Zamboni N., Seimbille Y., Hapfelmeier S.H., Stevanović A., Suarez-Zamorano N., Tarallo V., Fabbiano S., Montet X., Stojanović O., Rigo D. (2015). Gut Microbiota Orchestrates Energy Homeostasis during Cold. Cell.

[70] Bodogai M., O’Connell J., Kim K., Kim Y., Moritoh K., Gusev C.C., Vaughan K., Shulzhenko N., Mattison J.A., Lee-Chang C. (2018). Commensal bacteria contribute to insulin resistance in aging by activating innate B1a cells. Sci. Transl. Med..

[71] Kuczma M.P., Szurek E.A., Cebula A., Chassaing B., Jung Y.-J., Kang S.-M., Fox J.G., Stecher B., Ignatowicz L. (2020). Commensal epitopes drive differentiation of colonic T regs. Sci. Adv..

[72] Ansaldo E., Slayden L.C., Ching K.L., Koch M.A., Wolf N.K., Plichta D.R., Graham D.B., Xavier R.J., Moon J.J., Barton G.M. (2019). *Akkermansia muciniphila* induces intestinal adaptive immune responses during homeostasis. Science.

[73] Chen T., Chen X., Zhang S., Zhu J., Tang B., Wang A., Dong L., Zhang Z., Yu C., Sun Y. (2021). The Genome Sequence Archive Family: Toward Explosive Data Growth and Diverse Data Types. Genom. Proteom. Bioinform..

[74] Xue Y., Bao Y., Zhang Z., Zhao W., Xiao J. (2022). Database Resources of the National Genomics Data Center, China National Center for Bioinformation in 2022. Nucleic Acids Res..

