# Supplementary material for: Intestinal Microbial Diversity of Free-Range and Captive Yak in Qinghai Province
Source: Microorganisms. 2022 Mar 31;10(4):754. doi: 10.3390/microorganisms10040754 (PMC9028582; doi:10.3390/microorganisms10040754)
Supplement: Supplementary file 1 [file microorganisms-10-00754-s001.zip › Figure S1-S2.pdf]

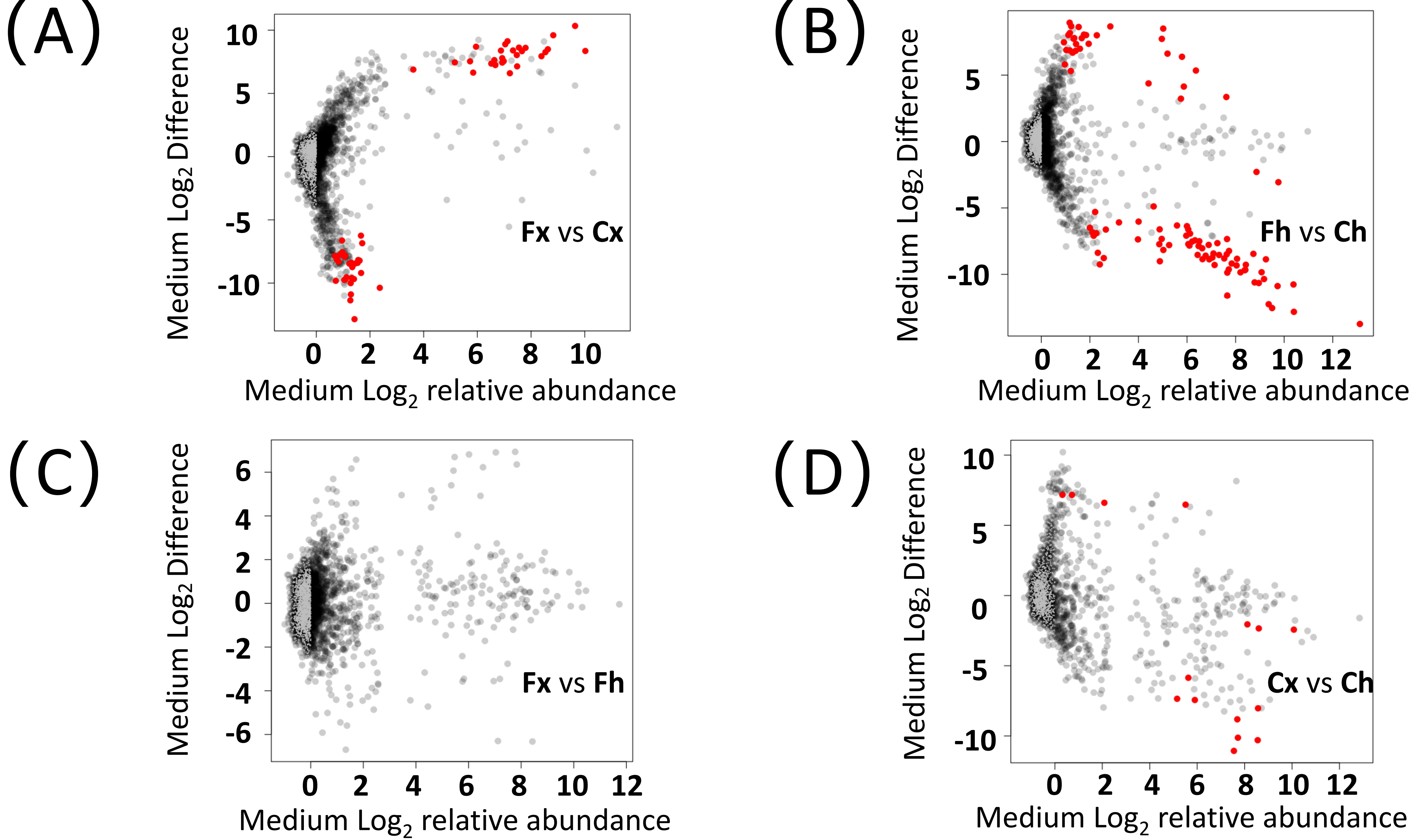

Figure S1. Differential abundance analysis using ADLEx2 method based on ASVs abundance. (A) Fx and Cx; (B) Fh and Ch; (C) Fx and Fh; (D) Cx and Ch. Red dots indicate ASVs with significant differences.

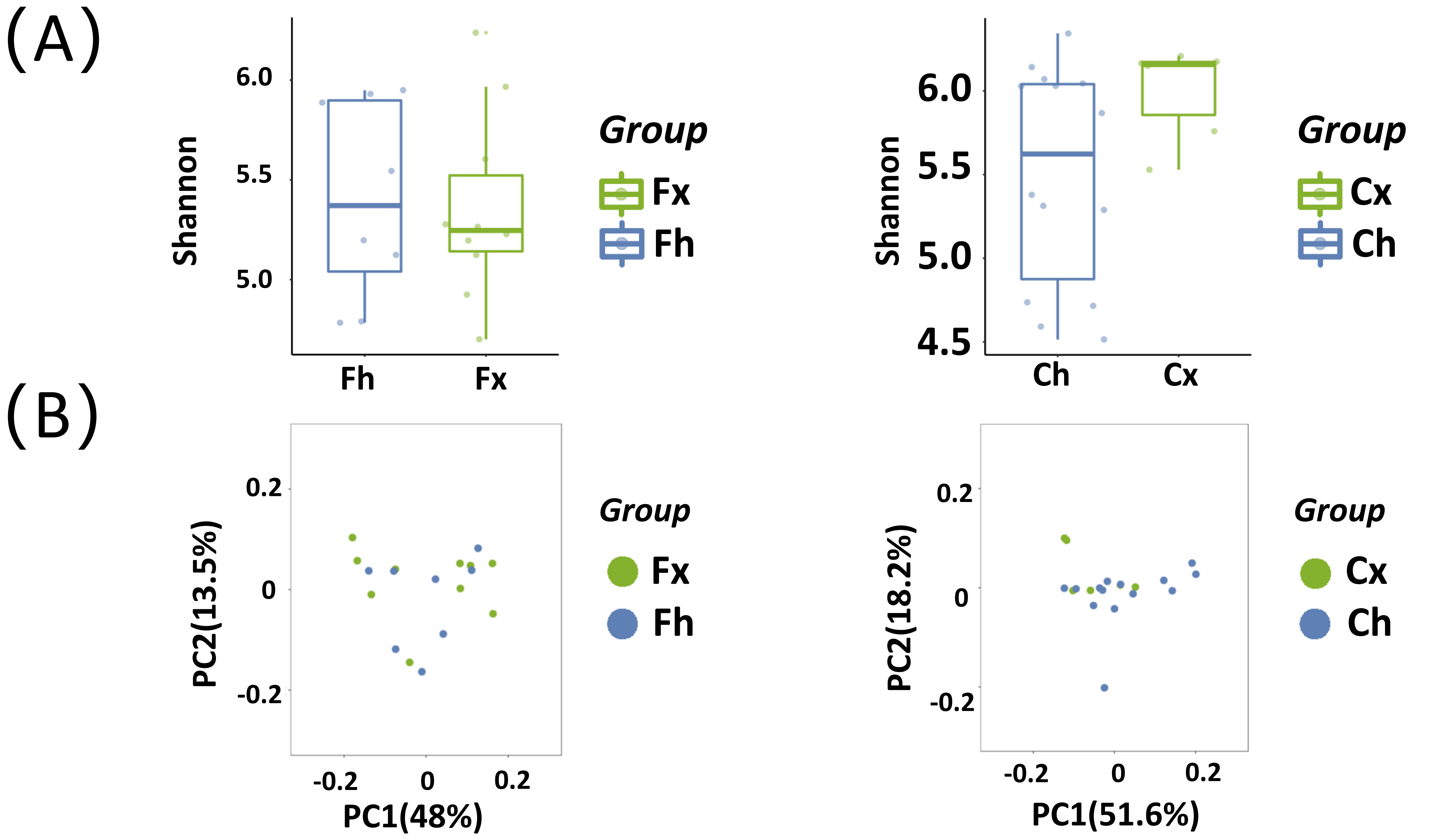

Figure S2. α and β diversity of yaks in the same feeding style. (A) Shannon index of yaks from different farms; (B) Principal component analysis (PCoA) based on unweighted UniFrac distance shows the distribution between samples from different farms.
