# Supplementary material for: Intestinal Microbial Diversity of Free-Range and Captive Yak in Qinghai Province
Source: Microorganisms. 2022 Mar 31;10(4):754. doi: 10.3390/microorganisms10040754 (PMC9028582; doi:10.3390/microorganisms10040754)
Supplement: Supplementary file 1 [file microorganisms-10-00754-s001.zip › Table S1-S4.pdf]

Table S1

Sample information of yaks.

No.	Feeding style	Region	Sample ID	Antibiotic treatment
1	Free-range	Xinghai county, Qinghai province	XF1	None
2	Free-range	Xinghai county, Qinghai province	XF5	None
3	Free-range	Xinghai county, Qinghai province	XF8	None
4	Free-range	Xinghai county, Qinghai province	XF9	None
5	Free-range	Xinghai county, Qinghai province	XF3	None
6	Free-range	Xinghai county, Qinghai province	XF6	None
7	Free-range	Xinghai county, Qinghai province	XF7	None
8	Free-range	Xinghai county, Qinghai province	XF10	None
9	Free-range	Xinghai county, Qinghai province	XF11	None
10	Free-range	Xinghai county, Qinghai province	XF12	None
11	Free-range	Haiyan county, Qinghai province	HF3	None
12	Free-range	Haiyan county, Qinghai province	HF4	None
13	Free-range	Haiyan county, Qinghai province	HF6	None
14	Free-range	Haiyan county, Qinghai province	HF7	None
15	Free-range	Haiyan county, Qinghai province	HF8	None
16	Free-range	Haiyan county, Qinghai province	HF9	None
17	Free-range	Haiyan county, Qinghai province	HF10	None
18	Free-range	Haiyan county, Qinghai province	HF14	None
19	Captive	Xinghai county, Qinghai province	XS1	None
20	Captive	Xinghai county, Qinghai province	XS2	None
21	Captive	Xinghai county, Qinghai province	XS3	None
22	Captive	Xinghai county, Qinghai province	XS4	None
23	Captive	Xinghai county, Qinghai province	XS5	None
24	Captive	Xinghai county, Qinghai province	XS6	None
25	Captive	Haiyan county, Qinghai province	HS1	None
26	Captive	Haiyan county, Qinghai province	HS2	None
27	Captive	Haiyan county, Qinghai province	HS3	None
28	Captive	Haiyan county, Qinghai province	HS4	None
29	Captive	Haiyan county, Qinghai province	HS12	None
30	Captive	Haiyan county, Qinghai province	HS13	None
31	Captive	Haiyan county, Qinghai province	HS14	None
32	Captive	Haiyan county, Qinghai province	HS5	None
33	Captive	Haiyan county, Qinghai province	HS6	None
34	Captive	Haiyan county, Qinghai province	HS7	None
35	Captive	Haiyan county, Qinghai province	HS8	None
36	Captive	Haiyan county, Qinghai province	HS9	None
37	Captive	Haiyan county, Qinghai province	HS10	None
38	Captive	Haiyan county, Qinghai province	HS11	None

Table S2

Sequence information of the sample.

No.	Sample	Raw reads	Filtered reads	ASVs
1	XF1	77293.7058954253*2	70490 *2	286
2	XF5	76498.1266757765*2	70717 *2	240
3	XF8	76083.3570507863*2	70094 *2	306
4	XF9	70418.7475894779*2	68441 *2	506
5	XF3	68335*2	65085 *2	944
6	XF6	70021.4281543477*2	68309 *2	646
7	XF7	75406.0307610242*2	70168 *2	311
8	XF10	76389.802590253*2	70453 *2	307
9	XF11	78025.6950488878*2	70976 *2	274
10	XF12	82057.130 9571032*2	71920 *2	238
11	XS1	70681.7036569505*2	68789 *2	356
12	XS2	71050.9367783478*2	68763 *2	307
13	XS3	71156.8953146654*2	68861 *2	419
14	XS4	71038.4675398457*2	68630 *2	324
15	XS5	71975.2082661832*2	69676 *2	428
16	XS6	70368.5695892826*2	68406 *2	256
17	HF3	71633*2	66059 *2	289
18	HF4	70997.3809523809*2	68964 *2	593
19	HF6	71106.9537752711*2	69105 *2	562
20	HF7	71441.2792092682*2	69103 *2	623
21	HF8	67026*2	60601 *2	293
22	HF9	77123.3796726441*2	70760 *2	232
23	HF10	69894.9599169218*2	68052 *2	457
24	HF14	79222.872883566*2	70621 *2	230
25	HS1	71362.447463706*2	69206 *2	343
26	HS2	70353.3640264654*2	68253 *2	273
27	HS3	70381.2170700929*2	68279 *2	395
28	HS4	70703.0168663615*2	68468 *2	381
29	HS12	70084.9546416625*2	68238 *2	380
30	HS13	76856.9654539165*2	69880 *2	195
31	HS14	70634.5509033215*2	68458 *2	425
32	HS5	71273.8525777323*2	69360 *2	398
33	HS6	70049.6185029864*2	68090 *2	313
34	HS7	70462.75444186*2	68268 *2	173
35	HS8	70241.9003517229*2	68293 *2	176
36	HS9	70785.7853377523*2	68512 *2	298
37	HS10	70415.7587655502*2	68038 *2	384
38	HS11	69914.3900310164*2	68100 *2	243

Table S3

Microbial sequence diversity and richness index of Yaks in Xinghai County.

Sample ID	Shannon	Simpson	ACE	Chao1
XF1	5.23	0.991	286.00	286.00
XF5	4.70	0.976	240.32	240.00
XF8	5.20	0.991	306.47	306.50
XF9	5.60	0.994	506.16	506.00
XF3	6.24	0.996	944.28	944.33
XF5	5.97	0.995	646.14	646.00
XF6	5.26	0.992	311.00	311.00
XF7	5.28	0.992	307.00	307.00
XF11	5.12	0.990	274.00	274.00
XF12	4.92	0.987	238.00	238.00
XS1	5.17	0.991	356.20	356.00
XS2	4.76	0.979	307.00	307.00
XS3	5.18	0.986	419.13	419.00
XS4	5.15	0.991	324.00	324.00
XS5	5.21	0.985	428.00	428.00
XS6	4.53	0.971	256.00	256.00
Mean ±SD	5.22±0.43	0.988±0.0071	384.36±184.09	384.30±184.10

Table S4

Microbial sequence diversity and richness index of Yaks in Haiyan County.

Sample ID	Shannon	Simpson	ACE	Chao1
HF3	5.20	0.991	289.00	289.00
HF4	5.89	0.995	593.96	598.00
HF6	5.93	0.996	562.96	567.00
HF7	5.95	0.996	623.30	623.25
HF8	5.12	0.990	293.63	293.50
HF9	4.79	0.982	232.00	232.00
HF10	5.54	0.994	457.15	457.00
HF14	4.78	0.986	230.00	230.00
HS1	4.87	0.980	343.00	343.00
HS2	4.29	0.963	273.18	273.00
HS3	5.03	0.977	395.00	395.00
HS4	5.03	0.983	381.30	382.00
HS5	5.07	0.983	380.00	380.00
HS6	4.31	0.954	195.00	195.00
HS7	5.34	0.990	425.00	425.00
HS8	5.04	0.984	398.00	398.00
HS9	3.74	0.834	313.00	313.00
HS10	3.51	0.934	173.00	173.00
HS11	3.59	0.939	177.75	177.50
HS12	4.38	0.963	298.00	298.00
HS13	5.14	0.985	384.15	384.00
HS14	3.72	0.936	243.17	243.00
Mean ±SD	4.83±0.73	0.970±0.0360	348.25±128.30	348.60±129.02
